# Investigation of the role of Ras and other signaling kinases on the regulation of pyruvate dehydrogenase complex activity

**DOI:** 10.1186/2049-3002-2-S1-P20

**Published:** 2014-05-28

**Authors:** Antonio Garcia-Trinidad, Nicky Whalley, Susan Critchlow

**Affiliations:** 1 Oncology, Astrazeneca, Macclesfield, UK

## 

Mutations in K-Ras commonly occur in colorectal, pancreatic and lung cancer, whereas mutations in BRAF are frequently found in melanomas. In past years, several studies have pointed to the role of Ras/Raf in the control of several metabolism pathways including glycolysis, mitochondrial respiration and glutamine metabolism [1-4]. The PDC complex is one of the central enzymes in the aerobic metabolism which converts the pyruvate produced during the glycolysis into Acetyl CoA used in the TCA cycle. In this work we show a pronounced sensitivity to PDHK-4 depletion in different mutant K-Ras cell lines, resulting in an increase of cell death and inhibition of cell growth in colorectal and lung tumor cell lines. Interestingly, cells expressing wild type K-Ras were signifinantly resistant to PDHK-4 depletion. This sensitivity in mutant Ras cells was correlated with a decrease in ERK phosphorylation, suggesting a possible inactivation in the Ras signaling pathway after PDHK-4 knock-down. Further studies will allow us to understand the biological mechanism by which Ras function is affecting the metabolic pathways involved in the regulation of PDC complex in cancer cells. Understanding the link between the PDC complex and Ras could be relevant to target cancer progression more effectively.
